# *Shb *deficient mice display an augmented T_H_2 response in peripheral CD4+ T cells

**DOI:** 10.1186/1471-2172-12-3

**Published:** 2011-01-11

**Authors:** Karin Gustafsson, Gabriela Calounova, Fredrik Hjelm, Vitezslav Kriz, Birgitta Heyman, Kjell-Olov Grönvik, Gustavo Mostoslavsky, Michael Welsh

**Affiliations:** 1 Department of Medical Cell Biology, Uppsala University, Uppsala, Sweden; 2 Olink Bioscience, Uppsala, Sweden; 3 Institute of Biophysics, Academy of Science of the Czech Republic, Brno, Czech Republic; 4 Department of Medical Biochemistry and Microbiology, Uppsala University, Uppsala, Sweden; 5 National Veterinary Institute, Uppsala, Sweden; 6 Department of Medicine, Section of Gastroenterology, and Center for Regenerative Medicine (CReM), Boston University School of Medicine, Boston, MA, USA

## Abstract

**Background:**

Shb, a ubiquitously expressed Src homology 2 domain-containing adaptor protein has previously been implicated in the signaling of various tyrosine kinase receptors including the TCR. Shb associates with SLP76, LAT and Vav, all important components in the signaling cascade governing T cell function and development. A *Shb *knockout mouse was recently generated and the aim of the current study was to address the importance of *Shb *deficiency on T cell development and function.

**Results:**

*Shb *knockout mice did not display any major changes in thymocyte development despite an aberrant TCR signaling pattern, including increased basal activation and reduced stimulation-induced phosphorylation. The loss of Shb expression did however affect peripheral CD4+ T_H _cells resulting in an increased proliferative response to TCR stimulation and an elevated IL-4 production of naïve T_H _cells. This suggests a T_H_2 skewing of the *Shb *knockout immune system, seemingly caused by an altered TCR signaling pattern.

**Conclusion:**

Our results indicate that Shb appears to play an important modulating role on TCR signaling, thus regulating the peripheral CD4+ T_H_2 cell response.

## Background

The primary aim of T cell development is to create a fully competent T lymphocyte population in the periphery, capable of quickly identifying pathogens yet non-responsive to self-tissue [1,2]. To maintain this delicate balance, the immature thymocytes are subjected to rigorous control, where signaling through the T cell receptor (TCR) is of utmost importance [3-5]. Mature, peripheral T cells are equally dependent on the TCR, as activation and effector cell development are decided by TCR signaling strength and duration [6-8].

Upon receptor engagement, a host of adaptor and effector proteins assemble at numerous phosphotyrosines within the cytoplasmic segment of the TCR signaling complex. Among the key players in this signaling system are the adaptors SLP76 [9,10] and linker for activation of T cells (LAT) [11,12], their association with the activated TCR and their subsequent phosphorylation that activates signaling through phospholipase C-γ (PLC-γ) [13], as well as Ras and the Rho family of GTPases [14,15], which in turn enable activation of more distal pathways such as the various variants of mitogen activated kinases (MAPKs) [16-18]. Loss of early signaling elements, such as LAT or SLP76 therefore has profound effects on T cell development and function, with an almost complete block at the very first thymocyte development step requiring TCR signaling, the β- selection, and with severely impaired peripheral responses as a consequence [11,19-21].

Shb is a widely expressed adaptor protein, known to associate with a variety of different tyrosine kinases, including receptors important for hematopoiesis in general and lymphopoiesis in particular, including the activated TCR, VEGFR- 2 and PDGFR [22-24]. In the case of TCR signaling, the SH2-domain of Shb binds to the ζ-chain of the CD3 complex, where it partakes in the LAT- SLP76 signaling complex [24,25]. Shb has been demonstrated to associate with both LAT and SLP76, and to facilitate the phosphorylation of numerous downstream signaling targets such as Vav-1 and PLC-γ, accordingly playing an important role in proper TCR signal transduction [24-26].

A *Shb *knockout mouse has recently been generated. No viable *Shb *knockout offspring could be generated on the C57Bl/6 background owing to an early embryonic defect. However *Shb *null mice were obtained on a mixed background (129Sv/C57Bl6/FVB) [27]. The animals display abnormalities in their reproduction, vasculature and glucose homeostasis [27-30]. We employed the *Shb *knockout to assess thymus and T cell function. Although early T cell development appears unaffected, we observe hyperproliferation and a skewing towards a T_H_2 response in peripheral T cells.

## Methods

### Animals

The generation of Shb knockout mice has been described previously [27]. The animals were bred on a mixed background (129Sv/C57Bl6/FVB). All experiments were approved by the local animal ethics committee at Uppsala University.

### Cell preparation

Freshly isolated thymi and spleens were gently crushed through a 70 μm cell strainer (BD Bioscience, Erembodegem, Belgium) and thereafter treated with Red cell lysis buffer (Sigma Aldrich, St. Lois, MO) in order to remove erythrocytes.

### Flow cytometry

For flow cytometry 1 × 10^6 ^cells in a final volume of 100 μl 1% BSA in PBS were stained with the following antibodies CD4-FITC, CD8-FITC, CD4-PE-Cy5, CD8- PE, CD44-PE-Cy5, CD25-PE, and CD62L-PE (all antibodies were purchased from eBioscience, Hartfield, UK). Flow cytometry was performed on a FACSCalibur (BD Bioscience) using CellQuest software (BD Bioscience, Franklin Lakes, NJ).

### TCR stimulation

Single cell suspensions with 1 × 10^7 ^thymocytes or 1 × 10^6 ^splenocytes in RPMI 1640 (Gibco, Paisley, UK) supplemented with 10% FBS and 50 μM β-mercaptoethanol (Sigma Aldrich) were stimulated with CD3-antibody (BD Bioscience) at a concentration of 10 μg/ml in 37°C. Thymocytes were stimulated for either 2 or 5 minutes whereas splenocytes were stimulated for 2 minutes.

### Immunoprecipitation and immunoblotting

Cell lysates were prepared by addition of lysis buffer (20 mMTris -HCl pH 7.8, 150 mM NaCl, 2 mM EDTA, 1%Triton X- 100, 2 mM PMSF, 10 μg/L aprotinin and 10 μg/L leupeptin). Immunoprecipitation was performed by incubating the samples with either α-phosphotyrosine antibody (Millipore, Watford, UK) or with Shb antibody [31] at 0.5 μg/ml or 5 μg/ml, respectively, for 1 hour at 4°C. This was followed by incubation with Protein A sepharose (GE Healthcare, Uppsala, Sweden) under the same conditions. Samples were washed 3 times in lysis buffer and resuspended in SDS-sample buffer.

Protein denaturation was achieved by boiling the samples for 5 minutes followed by separation on SDS- PAGE. Proteins were transferred to Hybond-P membranes (GE Healthcare) and subsequently blocked in 5% BSA over night at 4°C.*Immunodetection *The membranes were probed with the following antibodies: α-phosphotyrosine (Millipore), phospho-ERK (Cell Signaling Technology), PLCγ (Milipore), c-Cbl (BD Bioscience), Vav1 (Millipore), phospho-p38 (Cell Signaling Technology), ZAP70 (BD Bioscience), p38 (Cell Signaling Technology), Shb and ERK (Cell Signaling Technology). Immunodetection was performed using HRP-conjugated secondary antibodies (GE Healthcare) and ECL detection solution (GE healthcare) according to manufacturer's instructions. The autoradiographic film (GE healthcare) was afterwards exposed to the membrane for 2 seconds up to 2 minutes, depending on the strength of the signal.

### T cell purification, proliferation and cytokine production assay

Single cell suspensions of splenocytes were fractioned into either CD4+ or CD8+ cells or into naïve CD4+ cells using MACS magnetic microbeads (Miltenyi Biotec, Bergisch Gladbach, Germany), following the manufacturer's instructions. 5 × 10^5 ^CD4+ or CD8+ splenocytes were plated on 24-well plates precoated with 2.5 μg/ml α-CD3 and 3 μg/ml α-CD28 antibodies (BD Bioscience) in a volume of 1.5 ml F-DMEM (SVA, Uppsala, Sweden) supplemented with 10% FBS and 50 μM β-mercaptoethanol. Supernatants were harvested every 24 hours for 6 days and cytokine production was estimated by sandwich immunoassays using Gyrolab Bioaffy (Gyros Biotech, Uppsala, Sweden) following the manufacturer's instructions [32]. On the fourth day of stimulation, 10% of the cells were incubated with 1 μCi of ^3^H-thymidine (GE Healthcare) 4 hours, in order to estimate proliferative activity. The amount of radioactivity was thereafter determined with a Wallac 1409 scintillation counter (Perkin Elmer, Waltham, MA, USA). Naïve CD4+ cells were cultured under the same conditions as described above, but the cells were plated on 96-well plates at a concentration of 5 × 10^4 ^cells per well in 200 μl for 2 day cultures and 2.5 × 10^4 ^cells per well for 3 to 5 day cultures.

### Real-time reverse transcription- PCR

RNA was isolated from naïve CD4+ cells, at the indicated time points, using a RNAeasy mini kit (Qiagen, Solna, Sweden) following the manufacturer's instructions. Gene expression was quantified using the SYBR^® ^Green RT-PCR kit (Qiagen). PCR conditions were 50°C for 20 minutes and 95°C for 15 minutes, followed by 45 cycles with denaturation at 94°C for 15 seconds, annealing for 25 seconds at the various temperatures indicated in table 1 and extension at 72°C for 15 seconds. Primer sequences are listed in table 1. Transcription levels were normalized against β-actin. All PCR reactions were run on a Light Cycler (Roche Diagnostics, Basel, Switzerland) and C_T_-values were calculated using the accompanying software.

**Table 1:** PCR primer sequences and T_m _used for the RT-PCR analysis

Primers	Sequence (5'-3')	T_m_
Beta-actin 5'	CAC TAT TTG GCA ACG AGC GG	60°C

Beta-actin 3'	TCC ATA CCC AAG AAG GAA GGC	60°C

IL-2 5'	TTG AGT GCC AAT TCG ATG ATG	55°C

IL-2 3'	AGA TGA TGC TTT GAC AGA AGG CTA	55°C

IL-4 5'	CGG AGA TGG ATG TGC CAA AA	60°C

IL-4 3'	GCA CCT TGG AAG CCC TAC AG	60°C

IFN-γ 5'	CCT GGG GCC TAG CTC TGA	60°C

IFN-γ 3'	CAG CCA GGA ACA GCC ATG AG	60°C

GATA3 5'	CGA GAT GGT ACC GGG CAC TA	60°C

GATA3 3'	GAC AGT TCG CGC AGG ATG	60°C

Tbet 5'	TTC CCA TTC CTG TCC TTC ACC	60°C

Tbet 3'	TGC CTT CTG CCT TTC CAC AC	60°C

### Cell cycle analysis

The cell cycle status of naïve CD4+ T cells was analyzed at the indicated time points by adding 5-bromo-2-deoxyuridine (BrdU) (Sigma Aldrich) in a final conctration of 50 μM followed by a 2-hour incubation. The cells were subsequently fixed and permeabilized using the BD Cytofix/Cytoperm kit (BD Bioscience) following the instructions provided by the manufacturer. Cell cycle status was determined by staining with anti-BrdU-APC (Invitrogen Ltd, Paisley, UK) and 7-amino-actinomyocin D (7AAD) (eBioscience) followed by analysis on a FACSCalibur (BD Bioscience) using CellQuest software (BD Bioscience).

### Statistical analysis

Data is presented as mean ± Standard error of the mean (SEM). For comparison of difference between two groups with normal distributed data, unpaired Students t-tests were used unless otherwise stated. For paired comparisons, one wild type and one age and sex matched knockout sample was analyzed simultaneously, under identical conditions, and the wild type and the knockout values were set as one observation each for the comparison. All p-values less than 0.05 were considered statistically significant.

## Results

### Effect of Shb null allele on blood cell numbers

**Figure 1:**
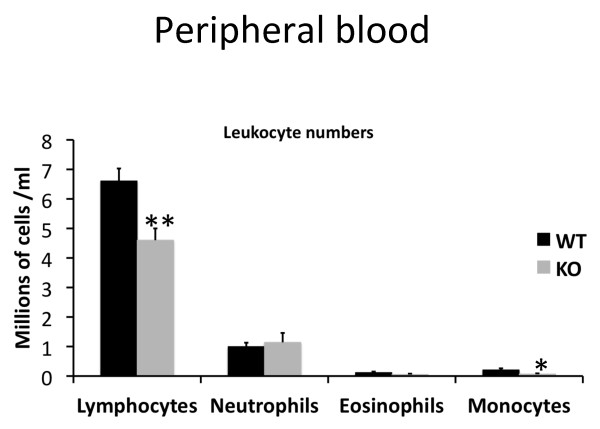
**Peripheral blood profile**. Peripheral blood was stained with May-Grünwald Giemsa in order to quantify leukocyte numbers (n = 8, mean ± SEM, ** denotes p < 0,01, * denotes p < 0,05 by Students t-test).

Since Shb has been demonstrated to interact with the TCR, but also with other receptors that are important for the development and function of the hematopoietic system it was of interest to determine whether the loss of Shb expression would result in any alterations in the number of blood cells. Hematocrit values and thrombocyte numbers appeared unchanged (data not shown), whereas both lymphocyte (WT 6.6 ± 0.42 million cells/ml; KO 4.6 ± 1.2 million cells/ml) and monocyte (WT 0.21 ± 0.05 million cells/ml; KO 0.07 ± 0.02 million cells/ml) numbers were decreased in the *Shb *knockout (lympocytes p < 0.01; monocytes p < 0.05) (Figure 1).

### Aberrant signal transduction in Shb knockout thymocytes

**Figure 2:**
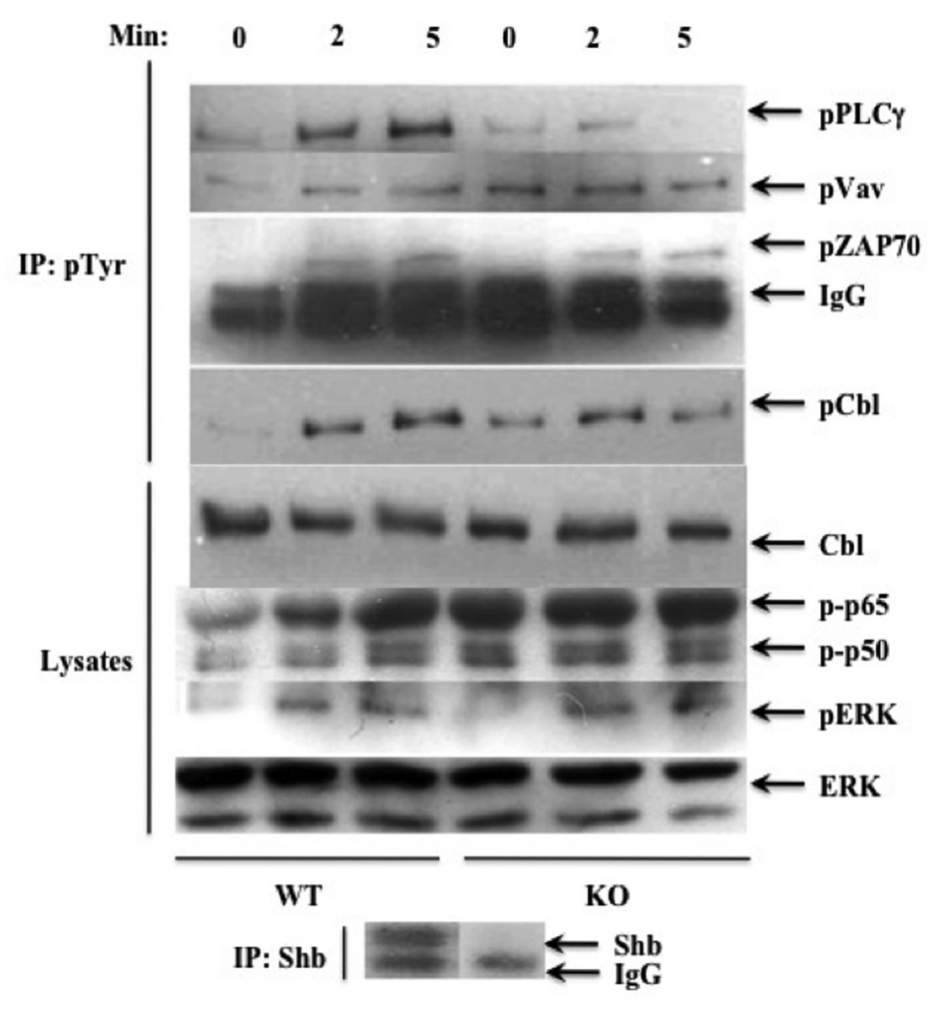
**Thymocyte stimulation response**. Thymocytes were stimulated with CD3-antibody for 2 and 5 minutes and analyzed by either immunoprecipitation with phosphotyrosine antibody followed by Western blot or by just performing Western blot on cell lysates. Blots were probed for phosphotyrosine, phospho-PLCγ, phospho-Vav, phospho-Zap70, phospho-Cbl, phospho-ERK, Cbl, ERK and Shb.

TCR signaling is of uttermost importance in thymocyte development as many of the survival and death signals are mediated through the receptor. Peripheral lymphocyte numbers may therefore be adversely affected by a disruption of signaling during early thymocyte development. To explore this possibility further, wild type and knockout thymocytes were stimulated for 2 and 5 minutes, followed by immunoprecipitation with phosphotyrosine antibody. This revealed alterations in important TCR signaling components. PLC-γ phosphorylation was lowered after both 2 and 5 minutes of stimulation in the *Shb *knockout (Figure 2) whereas Vav-1 and Cbl display increased basal phosphorylation levels in knockout thymocytes. Additionally, *Shb *knockout thymocytes fail to mount any stimulation-induced increase of Vav-1 and Cbl phosphorylation (Figure 2). A similar pattern is observed when the corresponding lysastes were probed for tyrosine phosphorylations; two major phosphotyrosine proteins of 65 and 50 kDa both display elevated basal phosphorylation levels but no stimulation effect (Figure 2). Other studies performed on *Shb *knockout endothelial cells and oocytes, have demonstrated elevated signaling in the absence of ligand-stimulation, suggesting this as being a feature in common in several signaling systems lacking Shb [28,30]. ZAP70 phosphorylation was unaffected by the absence of Shb, which is an expected finding since Shb is predicted to operate downstream of ZAP70 in the TCR signaling pathway (Figure 2). Moreover, extracellular signal regulated kinase (ERK) signaling, which has been implicated in positive selection [33,34], appeared similar in the two groups of cells after both 2 and 5 minutes of stimulation (Figure 2).

### Unaltered thymocyte development in the Shb knockout mouse

**Figure 3:**
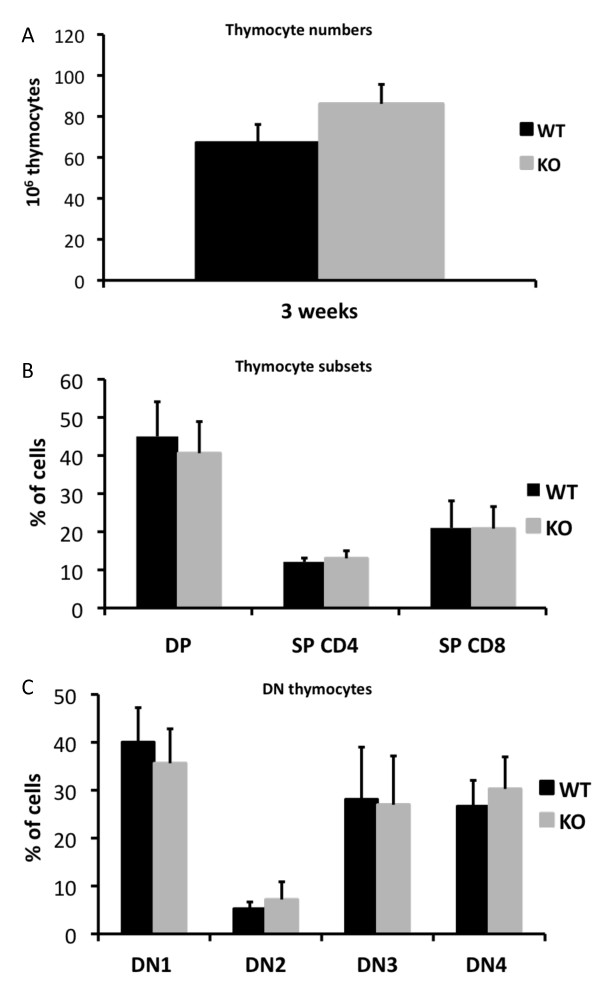
**Thymocyte maturation in mice 3 weeks of age**. (A) The number of thymocytes was determined by Bürker chamber counting (n = 9, mean ± SEM). (B) Thymocytes stained with fluorescent CD4 and CD8 antibodies and analyzed with flow cytometry (n = 9, mean ± SEM). (C) Thymocytes were isolated and stained with fluorescent antibodies directed against CD4, CD8, CD25 and CD44 and analyzed with flow cytometry (n = 9, mean ± SEM).

Since thymocytes exhibited a changed signaling pattern in response to TCR stimulation, thymi were isolated from mice 3 weeks of age, as thymocyte developmental activity is at a peak at this time [35]. Thymocyte numbers were determined, but no distinguishable differences between knockout and wild type animals were observed (Figure 3A). Even though there was no detectable deviation in thymocyte numbers, relative changes in the proportions of different developmental subsets in the thymus could cause the reduced numbers of lymphocytes in peripheral blood. Thymocytes were therefore stained for CD4 and CD8 in order to identify the different developmental stages [36,37]. However, *Shb *knockout animals displayed no statistically significant changes in the relative numbers of double positive (DP), single positive (SP) CD4+, and SP CD8+ cells (Figure 3B). Thymocytes were also analyzed for CD44 and CD25 expression to enable a distinction between the different double negative (DN) subsets [5], but the *Shb *knockout mice displayed no major differences in the DN1-4 populations (each given in percent of the total DN population) (Figure 3C).

### Shb knockout T_H _cells display altered signaling and proliferation

**Figure 4:**
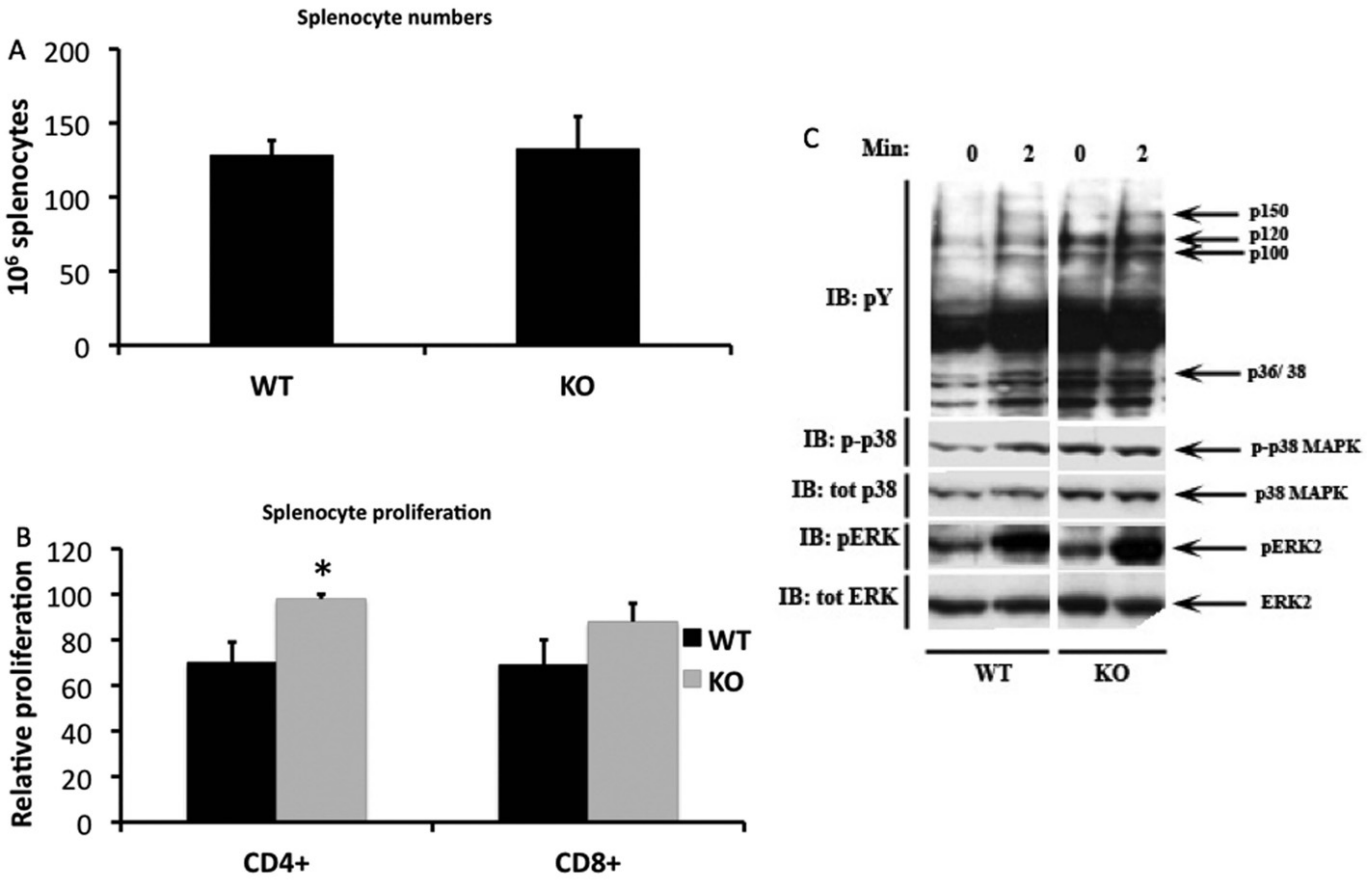
**Peripheral T cell signaling and proliferation**. (A) The number of splenocytes was estimated by counting cells in a Bürker chamber (n = 10, mean ± SEM). (B) Splenocytes were separated into CD4+ and CD8+ cells and stimulated for 96 hours with CD3- and CD28 antibodies. Radioactively labeled thymidine was added for the last 4 hours to estimate proliferative activity. The proliferation data is presented as a percentage of the highest value in each experiment that for CD4+ cells corresponds to 264326 ± 41432 and for CD8+ cells to 191290 ± 49852 DPM (n = 10, mean ± SEM, * denotes p < 0,05 by paired Students t-test). (C) CD4+ splenocytes were stimulated for 2 minutes with CD3 antibody and subsequently analyzed by Western blot. The samples were probed with phosphotyrosine,- phospho- p38-, phospho-ERK- and ERK antibodies.

Despite changes in the TCR response to stimulation observed in *Shb *knockout thymocytes, knockout mice exhibited no major changes in central T cell development. This prompted us instead to evaluate peripheral T cell function. We first analyzed absolute numbers of splenocytes, yet no detectable difference between knockout and wild type animals was noted (Figure 4A).

To further study the function of peripheral T cells, splenocytes were fractionated into CD4+ T_H _cells and CD8+ T_K _cells and stimulated with CD3 and CD28 antibodies for 96 hours after which proliferation was assessed by estimating ^3^H thymidine incorporation. When compared to the wild type cells, the *Shb *null CD4+ cells exhibited a modest proliferation increase in response to stimulation (WT 70 ± 9; KO 98 ± 2; p < 0.05) (Figure 4B). CD8+ *Shb *knockout cells also displayed a slightly elevated proliferative response, although this effect did not reach statistical significance (Figure 4B).

An increased proliferative rate is likely to be caused by changes in TCR signal transduction. T cell signaling was consequently examined by stimulating CD4+ T_H _cells with CD3 antibody for 2 minutes. In immunodetection with phosphotyrosine antibody the *Shb *knockout samples displayed an overall increased protein phosphorylation in the absence of stimulation (Figure 4C), a pattern similar to what was observed in thymocytes. More specifically there was a difference between wild type and knockout in the phosphorylation of a 150 kDa band under basal conditions as well as in stimulated cells (Figure 4C). In addition, bands of molecular weights 120 kDa, 100 kDa and 36/38 kDa exhibited a higher degree of phosphorylation in unstimulated Shb knockout samples, but phosphorylation levels did not appear to increase with stimulation.

In order to address the effect on other signaling pathways downstream of Shb, ERK and p38 MAPK activity was measured in the samples, as both are important for T cell survival and function. p38 MAPK stimulation was poor in the knockout, partly as a consequence of increased basal signal, whereas there was a clear increase in the amount of phospho-p38 MAPK in the wild type after stimulation (Figure 4C). ERK signaling on the other hand seemed normal (Figure 4C).

### T_H_2 skewing in Shb knockout T cells after stimulation

**Figure 5:**
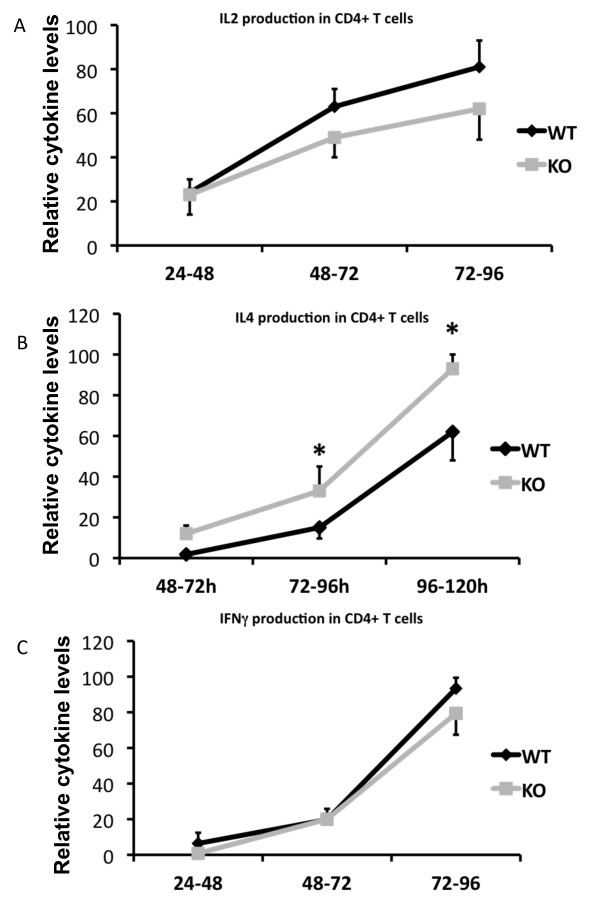
**T_H _cell profile in unfractionated CD4+ T lymphocytes in response to stimulation**. All cytokine data is presented as a percentage of the highest value (given in ng/ml) in each experiment and measured using the Gyros system. (A) The 100 percent IL-2 value corresponds to 9,2 ± 3,2 ng/ml (n = 8, mean ± SEM). (B) The 100 percent IL-4 value corresponds to 10 ± 3 ng/ml (n = 8, mean ± SEM, * denotes p < 0,05 by paired Students t-test). (C) The 100 percent IFN-γ value corresponds to 130 ± 38 ng/ml (n = 5, mean ± SEM).

Another important factor governing T cell proliferation is IL-2, a potent T cell mitogen [38]. To test whether altered cytokine production was also a part of the increased proliferation seen in *Shb *knockout T_H _cells, cytokine levels were monitored every 24 hours for up to 5 days. *Shb *knockout T cells were found not to produce more IL-2 than their wild type counterparts but rather less, although this difference failed to reach statistical significance (Figure 5A). IFN-γ and IL-4 production were also investigated, revealing increased levels of IL-4 in the *Shb *null samples at both 72 to 96 hours and at 96 to 120 hours (72-96 h WT 15 ± 5.4, KO 33 ± 12; 96-120 h WT 62 ± 14, KO 93 ± 7; 72-96 h p < 0.05, 96-120 h p < 0.05) (Figure 5B). IFN-γ levels were however similar in knockout and wild type (Figure 5C).

**Figure 6:**
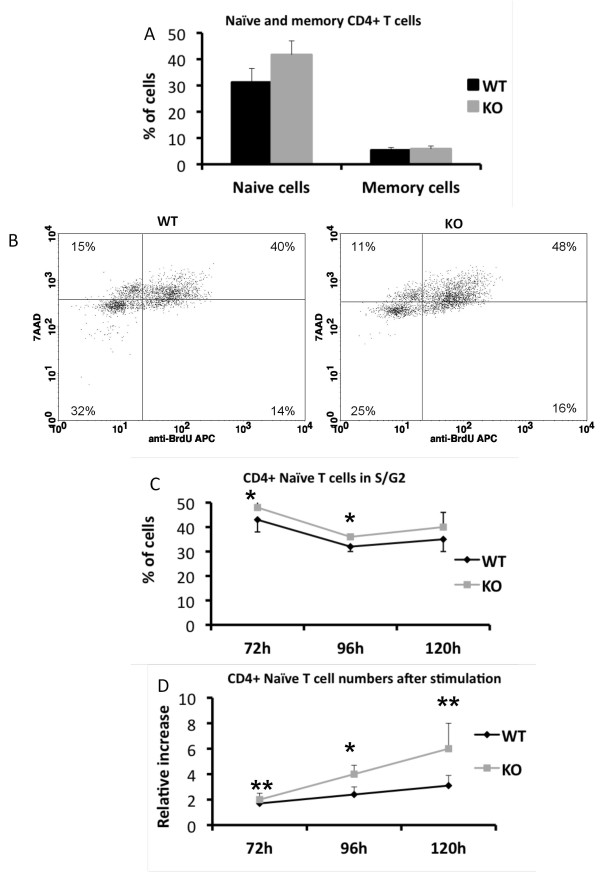
**Naïve and memory CD4+ T cell profile**. (A) Isolated splenocytes were stained for CD4, CD62L and CD44 in order to identify naïve and memory T cells (n = 6, mean ± SEM). (B, C) Naïve CD4+CD62L+ T cells were isolated and stimulated for 72, 96 or 120 hours followed by 2 hours of BrdU-pulse labeling. Cell cycle status was subsequently assessed based on BrdU and 7AAD staining. Plots represent typical experiment (n = 6, mean ± SEM, * denotes p < 0.05 by paired Students t-test). (D) The numbers of stimulated naïve CD4+CD62L+ T cells were determined at the indicated time points and related to the initial numbers of cells plated to determine relative increase in cell numbers (n = 6, mean ± SEM, * denotes p < 0.05, ** denotes p < 0.01 by paired Students t-test).

IL-4 is the hallmark cytokine of a T_H_2 response and increased IL-4 levels is indicative of a bias towards T_H_2 driven immune response [39]. A T_H_2 skewing in combination with hyperproliferation often coincides with an increase in the proportion of peripheral memory T cells [21,40]. Memory and naïve T cells are distinguished by their differential expression of CD44 and CD62L [41,42], *Shb *knockout and wild type T cells were consequently stained for these markers in order to determine if knockout mice had higher levels of memory cells. However, no difference in the proportions of memory and naïve T cells was noted between knockout and wild type animals (Figure 6A).

To further confirm that *Shb *knockout CD4+ T_H _cells have a tendency to develop a type 2 cytokine response, naïve CD4+CD62L+ T cells were isolated and stimulated. BrdU incorporation and 7AAD staining revealed that naïve knockout T cells appear to progress faster through the cell cycle when stimulated, as demonstrated by the modest but consistent increased percentage (p < 0.05) of *Shb *knockout cells in the later stages of the cell cycle at 72 and 96 hours of stimulation (72 h WT 43 ± 5%, KO 48 ± 4%; 96 h WT 32 ± 2%, KO 36 ± 1%) (Figure 6B and 6C). Additionally, when cell numbers were determined at the given time points the relative increase in cell numbers were higher in *Shb *knockout samples compared to the wild type (72 h WT 1.7 ± 0.5 fold increase, KO 2.4 ± 0.5 fold increase; 96 h WT 2.4 ± 0.6 fold increase, KO 4.0 ± 0.7 fold increase; 120 h WT 3.1 ± 0.8 fold increase, KO 6.0 ± 2.0 fold increase, all compared with the starting number of cells; 72 h p < 0.01, 96 h p < 0.05, 120 h p < 0.01).

**Figure 7:**
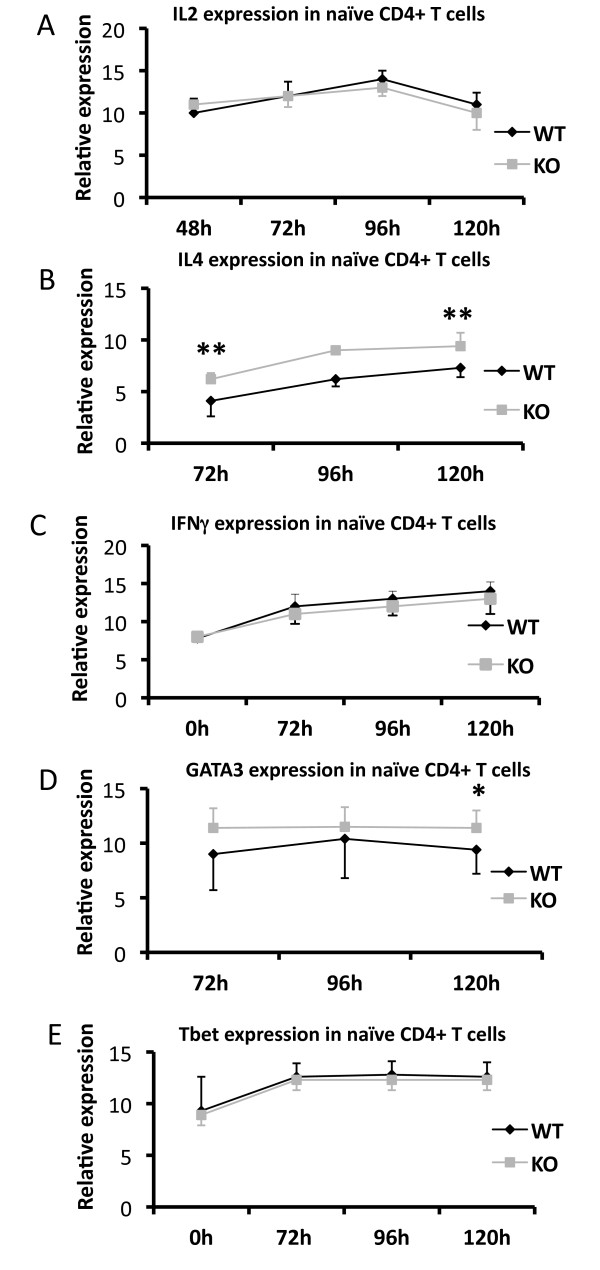
**T_H _cell gene expression profile in unfractionated naïve CD4+CD62L+ T lymphocytes in response to TCR stimulation**. (A, B, C) Transcript levels of cytokines IL-2, IL-4 and IFN-γ were determined by semiquantitative RT-PCR and normalized against the expression of the house keeping gene β-actin. Data is presented as 20 minus the mean of the normalized C_T _values ± SEM. (n = 6, ** denotes p < 0,01 by paired Students t-test). (B, C, D) Relative expression of the transcription factors GATA3 and T-bet were estimated through a semiquantitative RT-PCR and normalized against the expression of the house keeping gene β-actin. GATA3 is presented as 30 minus the mean of the normalized C_T _values and Tbet as 20 minus the mean of the normalized C_T _values ± SEM (n = 6, * denotes p < 0,05 by paired Students t-test).

Since *Shb *null naïve T cells also exhibited a lymphoproliferative phenotype we decided to assay cytokine production in CD4+CD62L+ T cells after TCR stimulation. Levels of mRNA revealed that IL-2 was produced in similar amounts in knockout and wild type cells (Figure 7A). IL-4 gene expression was elevated at all recorded time points but only reached statistical significance at 72 and 120 hours (72 h WT 4.1 ± 0.68, KO 6.2 ± 0.40; 92 h WT 6.2 ± 0.90, KO 9.0 ± 1.3; 120 h WT 7.3 ± 1.2; KO 9.4 ± 1.5, 72 h p < 0.05, 120 h p < 0.05). The differences in C_T_-values correspond to an increase of the *Shb *KO IL-4 mRNA levels compared with WT to 470 ± 110% and 230 ± 20% at 72 and 120 h, respectively (Figure 7B). IFN-γ levels were on the other hand similar in knockout and wild type samples (Figure 7C).

The differentiation of a newly activated T cell into a T_H_2 or a T_H_1 cell is governed by the transcription factors GATA3 and T-bet, respectively [43,44]. To further ascertain whether the *Shb *knockout displays a T_H_2 skewing the expression of these transcription factors was studied. The lack of *Shb *resulted in an elevated expression of GATA3 after 120 hours of stimulation (120 h WT 4.4 ± 2.2, KO 6.4 ± 1.6; p < 0.05) (Figure 7D) whereas T-bet levels appeared unaffected (Figure 7E). The altered C_T_-value corresponds to a 17.7 ± 8.5 fold increase in GATA3 mRNA in the *Shb *KO.

## Discussion

We have previously demonstrated the involvement of the adaptor protein Shb in TCR signal transduction in Jurkat cells. The current study expands these observations by examining T cell development and T cell function in a *Shb *knockout mouse. Our data establish that Shb is dispensable for thymocyte development but that it exerts effects on peripheral CD4+ T cell signaling. Consequently, unfractionated CD4+ and purified naïve T cells proliferate at a higher rate. An increased number of memory T cells is most probably not the cause of the *Shb *knockout phenotype since no difference in the proportions of memory and naïve CD4+ T cells was noted and the hyperproliferative effect was also observed on purified naïve T cells. The aberrant TCR signaling is a more likely candidate for the observed elevation in proliferation.

*Shb *knockout CD4+ T lymphocytes displayed accelerated levels of tyrosine phosphorylation under basal conditions. Several signaling components, putatively Vav-1, LAT and p38 MAPK, were markedly more phosphorylated in the absence of TCR activation. Stimulation-independent signaling has been implicated as an important factor in determining TCR signaling responsiveness [45,46]. For instance, microRNA-181, a negative regulator of several protein phophatases involved in TCR signaling, causes increased basal signaling and a lowered activation threshold when over-expressed [46]. The elevated basal phosphorylation of important TCR pathway targets in *Shb *knockout CD4+ T cells could make them prone to respond quicker and more vigorously to a given stimulus than their wild type counter parts.

Even though modifications in basal signaling might in part explain the high proliferative rate displayed by *Shb *knockout mice, the phosphorylation pattern of LAT is also important to take into consideration. Shb and LAT association, in response to TCR activation, has been demonstrated in Jurkat cells [24], and mice expressing the LAT^Y136F ^mutant show a phenotype reminiscent of the one present in the *Shb *knockout, with T_H_2 skewing and lymphoproliferation [21,40]. As already mentioned, a product probably corresponding to LAT (p36/38) exhibited a high level of basal phosphorylation in *Shb *null T cells, without becoming additionally phosphorylated by stimulation. LAT was originally identified as a key adaptor protein in the TCR signaling cascade, responsible for signals essential to T cell activation. Recent studies have revealed that LAT is not only a mediator of positive signals but also an important negative regulator of TCR signaling [47]. The defective stimulation-induced LAT phosphorylation displayed by *Shb *knockout T lymphocytes could result in a suboptimal assembly of the LAT signalosome. Consequently, negative feedback loops acting on the TCR machinery might be affected augmenting the proliferative response. On the other hand, LAT^Y136F ^mice have increased numbers of CD4+ memory cells [21,40], and as above mentioned, no such increase could presently be detected in the *Shb *knockout.

The changes in peripheral CD4+ signaling resemble those observed in the *Shb *knockout thymus. In *Shb *knockout thymocytes Vav-1 displayed increased phosphorylation under basal conditions without any signs of further amplified phosphorylation after TCR stimulation. Cbl is a ubiquitin ligase that exerts a role in down- regulating ZAP70 activity upon TCR stimulation [48]. Since no difference in ZAP70 activity was noted upon TCR stimulation between wild type and *Shb *knockout, it seems unlikely that the altered Cbl phosphorylation pattern observed plays any major role in affecting the *Shb *knockout T cell phenotype. Instead the changes in Cbl phosphorylation are probably a mere reflection of the overall effects of Shb deficiency on the TCR signaling complex.

T cell proliferation is dependent on TCR signaling that in turn indirectly promotes cell division by activation of IL-2 transcription [38]. IL-2 production was, however, normal in knockout cells despite the appearance of an increased responsiveness to TCR stimulation excluding this as an explanation for the *Shb *knockout hyper-responsiveness.

In addition, Shb appears to affect more than proliferation, since the cytokine production was also different from that observed in wild type T cells. Upon activation, CD4+ T cells have the choice of maturing into different classes of effector cells, each characterized by their own cytokine profile. T_H_1 and T_H_2 cells are the two major subsets, responsible for cellular and humoral immune responses, respectively [39,49]. Signaling from the TCR and cytokine receptors are of great importance in the development of these effector cell responses. Alterations in the activities of targets downstream of the TCR might therefore also affect cytokine production. Vav-1 has for instance been demonstrated as an important factor in IL-4 production and the generation of a T_H_2 response. Vav-1 knockout mice preferentially develop a T_H_1 response and Vav-1 in synergy with protein kinase C-Θ (PKC-Θ) has been implicated in the promotion of IL-4 transcription [50,51]. As already noted *Shb *knockout thymocytes as well as CD4+ T cells exhibited a slight alteration in their Vav-1 activation. The increased basal activity of Vav-1 in knockout cells may alter the intracellular signaling conditions in favor of a T_H_2 response.

Moreover, the development kinetics of T_H_1 and T_H_2 cells are quite different. The hallmark cytokine of T_H_1 cells, IFN-γ is produced within hours of activation. Transcripts from typical type 2 cytokines such as IL-4 are on the other hand detected at the earliest on day 2 of stimulation in any significant amounts and a full-fledged T_H_2 response can take weeks to develop [52,53]. The main reason for the slow development of T_H_2 cells is thought to be the extensive chromatin remodeling that is required to fully open the *il4 *gene locus for transcription [54,55]. A critical part of the remodeling process is cellular proliferation and it has even been suggested that a certain number of cell divisions are required before IL-4 transcription occurs [56]. Naïve and unfractionated CD4+ T cells from *Shb *null mice produce IL-4 more promptly after stimulation. Since absence of Shb appeared to lead to an increased cell division rate and faster cell cycle progression it may well result in a more accessible *il4 *locus thus contributing to a slight T_H_2 skewing in the immune system of *Shb *knockout mice.

## Conclusion

In the present work, we observe that CD4+ naïve T lymphocytes lacking Shb exhibit increased proliferation due to alterations in important TCR signaling pathways also resulting in a bias towards developing a T_H_2 cytokine response. Further studies of the effects of Shb on the immune system may therefore prove useful in the elucidation of T_H_2 driven pathologies such as allergies.

## Competing interests

The authors declare that they have no competing interests.

## Authors' contributions

KG, GC, FH, VK, GM and MW performed the experiments. KG, BH, KOG, GM and MW participated in the experimental design and KG, GM and MW analyzed the results. All authors have read the study and agreed to its content.
